# Unraveling disparate roles of organisms, from plants to bacteria, and viruses on built cultural heritage

**DOI:** 10.1007/s00253-023-12423-5

**Published:** 2023-02-23

**Authors:** Patricia Sanmartín, Pilar Bosch-Roig, Domenico Pangallo, Lucia Kraková, Miguel Serrano

**Affiliations:** 1 GEMAP (GI-1243), Departamento de Edafoloxía e Química Agrícola, Facultade de Farmacia, Universidade de Santiago de Compostela, 15782 Santiago de Compostela, Spain; 2 CRETUS, Universidade de Santiago de Compostela, Santiago de Compostela, Spain; 3 Instituto Universitario de Restauración del Patrimonio, Dpto. Conservación y Restauración del Patrimonio, Universitat Politècnica de València, 46022 Valencia, Spain; 4 Institute of Molecular Biology, Slovak Academy of Sciences, Dúbravská cesta 21, 845 51 Bratislava, Slovakia; 5 Caravella, s.r.o., Tupolevova 2, 851 01 Bratislava, Slovakia; 6 Department of Botany, Faculty of Pharmacy, University of Santiago de Compostela, 15782 Santiago de Compostela, Spain

**Keywords:** Biocontrol agents (BCAs), Biotreatment, Ecological factors, Species adaptation, Species role in ecosystems, Stone-built heritage

## Abstract

**Abstract:**

The different organisms, ranging from plants to bacteria, and viruses that dwell on built cultural heritage can be passive or active participants in conservation processes. For the active participants, particular attention is generally given to organisms that play a positive role in bioprotection, bioprecipitation, bioconsolidation, bioremediation, biocleaning, and biological control and to those involved in providing ecosystem services, such as reducing temperature, pollution, and noise in urban areas. The organisms can also evolve or mutate in response to changes, becoming tolerant and resistant to biocidal treatments or acquiring certain capacities, such as water repellency or resistance to ultraviolet radiation. Our understanding of the capacities and roles of these active organisms is constantly evolving as bioprotection/biodeterioration, and biotreatment studies are conducted and new techniques for characterizing species are developed. This brief review article aims to shed light on interesting research that has been abandoned as well as on recent (some ongoing) studies opening up new scopes of research involving a wide variety of organisms and viruses, which are likely to receive more attention in the coming years.

**Key points:**

• *Organisms and viruses can be active or passive players in heritage conservation*

• *Biotreatment and ecosystem service studies involving organisms and viruses are shown*

• *Green deal, health, ecosystem services, and global change may shape future research*

## Introduction

The study of organisms with an interacting role with cultural heritage, especially those with high metabolic activity and resilient to changes in environmental conditions, either by their growth form or their potential for genetic and physiological adaption, has become a research hotspot to advance fundamental knowledge to address the natural imbrication of different types of conservation problems that is usually present in built cultural heritage. It has been suggested that future research should focus on metabolically active microorganisms and biochemical reactions (Liu et al. 2022a), such as the transformation of metal compounds into metal oxalates (biopatination) by fungi and the consolidation of biofilms by extracellular polymeric substances (EPS) produced by bacteria (Joseph 2021). Bacteria can also induce precipitation of silicate, phosphate, sulfide, oxide, and above all, carbonate minerals (bioconsolidation), resulting from the interactions between metabolic by-products on substrate surfaces and the surrounding environment (Hoffmann et al. 2021). The precipitation of biominerals is promoted by high pH, as well as by high concentrations of calcium and dissolved inorganic carbon and the availability of nucleation sites (Timoncini et al. 2022). Previous studies have also confirmed microbial-rock interactions involving CO_2_ uptake or release processes; e.g., the development of actinobacterial biofilms on cave walls promotes uptake of CO_2_, dissolution of the rock, and production of calcite crystals during periods of low humidity and/or CO_2_ levels (Martín-Pozas et al. 2020). Methanotrophic bacteria consume CH_4_ in caves, produce methanobactins (bioactive compounds with high affinity for metal ions), and exhibit antibiotic activity against Gram-positive bacteria (Martín-Pozas et al. 2020). Although there has been an increase in these types of studies on biotreatments in heritage field, other research that produced promising results more than 10 years ago was unfortunately abandoned, e.g., studies on the use of phages as natural antagonists of bacteria and their use in bioremediation to remove algal growth from stone-built heritage (Klaassen 2005; May et al. 2009). The reasons for abandonment could be related to the lack of effectiveness of the treatment in the field, as well as the difficulty for its implementation in relation to costs and method of application.

Regarding the resilience of organisms and their ability to compete, Alexander Fleming warned, shortly after he discovered penicillin, of the problems that abusive use of the compound could bring about and that bacteria could become resistant to the antibiotic (Marshall and McMurry 2005). In a similar way, repeated application on the same target area of heritage interest of non-lethal concentrations of the biocide benzalkonium chloride (probably the most widely used quaternary ammonium compound in cultural heritage) over time (cleaning campaigns conducted during the 1980s, 1990s, 2000s) has probably driven a significant reduction in microbial sensitivity to biocides, creating tolerant and resistant species (Pinna 2022). This situation led to the search for novel biocidal solutions being heightened, so that at the beginning of the 1990s, there were about two dozen of antibacterial biocides on the market, and by 2005, they were 1000 or more (Marshall and McMurry 2005). The search also promoted a shift towards green biocides, for achieving more energy-, time-, and cost-efficient production and consumption processes, as promising alternatives to their industrial counterparts (see, e.g., Caldeira 2021).

Furthermore, many paradigms are changing rapidly; e.g., lithobionts are now considered to have a dual role in biodeteriorative and bioprotective effects, which sometimes take place simultaneously (Favero-Longo and Viles 2020, and references therein). Climbing plants and chromatic alterations on stone surfaces caused by biological growth were previously considered to enhance the aesthetic value of historical buildings and ruins (Martines 1983; Guillitte 1993). However, in later times, particularly in the late 1980s and 1990s, the presence of surface-colonizing organisms tended to be viewed as negative and associated with physical and chemical degradation, fouling, soiling, and loss of identity of the object. This led to the complete eradication of all organisms on the surfaces of the built heritage being favored and promoted the presence of “stone cities” (karst landscape), rather than “green cities.” However, this situation was countered by bioprotection studies, understood as “the positive ways in which organisms growing on the surfaces of rocks and building materials protect the surface from other processes of weathering and erosion” (Carter and Viles 2005; Sanmartín et al. 2021). Bioprotection roles include umbrella effects against rain droplets, wind abrasion, and solar radiation; the development of resistant outer layers, which prevent erosion or weathering due to pollutants; stabilization caused by filamentous actinobacteria, fungi, and lichens; and consolidation by secondary minerals, precipitates, and EPS-chelates (Liu et al. 2022b). Some bioprotective organisms are material binders and act as good substitutes for cementitious materials.

Likewise, long-term protection and the permanence of active organisms should be considered; e.g., live *Pseudomonas stutzeri* cells should be allowed to remain in treated areas of stonework to continue their nitrate biocleaning function (Pavlović et al. 2022).

**Fig. 1:**
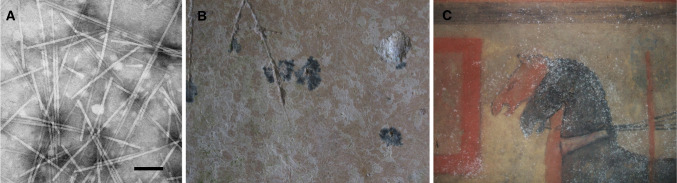
**A** Negative staining of a purified tobavirus product (in the form of rods) isolated in the laboratory. Bar = 100 µ. Photo: Vicente Medina. **B** Violet stains caused by *Streptomyces* sp. bacterium in the Circular Mausoleum, Roman Necropolis of Carmona, Spain. Photo: Cesáreo Saiz-Jiménez. **C** Bacteria of the order *Rhizobiales* on the paintings in Tomba del Colle (Etruscan tomb, near Chiusi, Italy). Photo: Cesáreo Saiz-Jiménez

This mini-review covers some case studies involving cultural heritage which have attempted to understand the active roles of various viruses and organisms (Fig. 1), ranging from bacteria to vascular plants, shedding light on interesting research which will probably receive more attention in the coming years.

## QAC-tolerant and QAC-resistant microorganisms

According to current European Union regulations (Aa.Vv. 2012), a biocide is defined as “any substance or mixture, in the form in which it is supplied to the user, consisting of, containing or generating one or more active substances, with the intention of destroying, deterring, rendering harmless, preventing the action of, or otherwise exerting a controlling effect on, any harmful organism by any means other than mere physical or mechanical action.” Examples of biocides include disinfectants, preservatives, antiseptics, pesticides, herbicides, fungicides, and insecticides. Biocides can be classified in several groups according to their functional chemical groups or their targets of action (Gnanadhas et al. 2013).

Biocides based on quaternary ammonium compounds (QACs), used as pesticides as well as biocides and with benzalkonium chloride (BAC) and didecyldimethylammonium chloride (DDAC) as main referents, have been used to treat microbial colonization on cultural heritage objects and are still widely used, mainly in stone conservation (Lo Schiavo et al. 2020). QACs target the microbial cell membrane, and electrostatic interactions between the positively charged QAC head and the negatively charged microbial cellular membrane are followed by permeation of the QAC side chains into the intramembrane region, ultimately leading to leakage of cytoplasmic material and cellular lysis (Gnanadhas et al. 2013).

Microorganisms on the surface of cultural heritage objects form communities called subaerial biofilms (SABs, Gorbushina 2007). Several microorganisms have the ability to secrete extracellular polymeric substances (EPS), which generally gives biofilms their adhesive properties, as well as reducing desiccation and providing organisms with a favorable microenvironment (Flemming 2016). Lichens (especially crustose lichens) and mature biofilms are extremely difficult to remove because they penetrate the surface even for a few millimeters adhering firmly to the substrate and also to eradicate because the presence of EPS and cells of low metabolic activity interfere with the action of biocides reducing their efficacy (Pinna 2022). Nevertheless, the application of biocide treatments with QACs as the last stage in the cleaning treatment of cultural heritage stone surfaces, to maintain the results longer, is a standard practice. But, what occurs at later stages? Does the colonization arise again? Is it the same as before? How does it affect conservation of the material, especially in the long term?

It has been shown that re-colonization of the surface might occur after the application of QACs, particularly on porous substrates (highly bioreceptive, i.e., substrates with high capacity to be colonized by living organisms) and areas that remain humid for long periods (Sanmartín et al. 2021; Pinna 2022). Re-colonization can be favored by new microorganisms colonizing the surface and using the residues of dead biomass and biocide compounds as nutrients, or by indigenous microorganisms, previously present on the surface and exposed to the biocide, which has developed resistance and now increase their presence (Pinna 2017; Sanmartín and Carballeira 2021). Some researchers have described the re-colonization of outdoor objects by lichens after treatment with different biocides, such as Biotin T®, Biotin R®, tributyltin oxide, dibutyltin dilaurate, and copper nanoparticles, identifying substrate bioreceptivity and climatic conditions as the main factors involved in the revitalization of viable fungal, green algal, and cyanobacterial cells (e.g., De los Ríos et al. 2012; Pinna et al. 2018). Other examples include algae and cyanobacteria, which were able to re-colonize the surface of stone and majolica-glazed tiles (Coutinho et al. 2016; Pfendler et al. 2018). Fungi can also be considered as newly emerging re-colonizers of cultural and artistic stone surfaces treated with biocides (Isola et al. 2022). The most famous and representative example of re-colonization by fungi is probably that of the Lascaux Caves (SW France) after years of successive treatments with biocides and antibiotics, following the first occurrence (between 1955 and 1960) of “the white disease” and “the green disease” or “Maladie Verte” (in French). The latter, caused by the unicellular alga *Bracteacoccus minor* (*Chlorophyta*, *Chlorococcales*), was first treated (summer 1963) with a mixture of antibiotics, i.e., penicillin, streptomycin, and kanamycin, which were dissolved in sterile double distilled water and delivered to the environment in the form of an aerosol, followed by a biocide treatment with an aqueous formaldehyde solution (Lefèvre 1974). Between 2001 and 2003, benzalkonium chloride (50%) was sprayed on the cave surfaces to eradicate (albeit not successfully) the white fungus *Fusarium solani*, which had begun to appear. During the same period, *Pseudomonas fluorescens* also appeared and was treated with antibiotics (streptomycin sulfates and polymyxin). Between 2007 and 2011, more than 30 strains of fungi were isolated from the caves. Of these, two belonged to new species of melanized fungi, described as *Scolecobasidium* (= *Ochroconis*) *lascauxensis* and *Scolecobasidium* (= *Ochroconis*) *anomala*, and also human opportunistic fungi such as *Exophiala castellanii* and *Exophiala moniliae* (Saiz-Jimenez et al. 2012).

The long-term effects of QACs on the substrate are still poorly understood, neither in relation to its own stability (i.e., no physical–chemical degradation) over time, nor in relation to how this affects re-colonization. Nevertheless, it has been shown that the repeated application of QACs is not advisable because this can lead to the development of some species biocide-tolerant microorganisms, versus whom QACs become ineffective. In this sense, bacteria are capable of re-colonizing surfaces previously treated with QAC biocides (Urzì et al. 2016; Kakakhel et al. 2019; Lo Schiavo et al. 2020; Sanmartín and Carballeira 2021). Moreover, there are some evidences that bacteria have developed mechanisms to resist QACs. Bacterial mechanisms of resistance to QACs have begun to be studied as the resistant bacteria may represent a threat to public health (Meade et al. 2021). In addition, a dangerous relationship between resistance to QACs and to antibiotics was noted in bacteria and found to be due to the genetic linkage of genes for QAC tolerance and antibiotic resistance (Mulder et al. 2018). Genes conferring reduced susceptibility to QACs are called *qac*. These bacterial genes encode efflux pumps, which are capable of expelling many QAC structures from bacterial cells. This serves to decrease the susceptibility of bacteria to QAC disinfectants. The *qac* genes are commonly found in class 1 integrons, which can occur as part of mobile genetic elements such as plasmids and transposons. Consequently, *qac* genes can be transferred horizontally via mobile genetic elements to other bacteria. This process can occur at the same time as the transfer of other antibiotic-resistant genes (Mulder et al. 2018; Vijayakumar and Sandle 2019). Research focused on detecting these types of genes in cultural heritage objects has only been attempted recently (He et al. 2022), and members of the genera *Pseudonocardia*, *Sphingomonas*, and *Streptomyces* have been recognized as major hosts of genes conferring resistance to antibiotics and biocides.

The literature search conducted for this review showed that very few studies have focused on unveiling the mechanisms of QAC biocide resistance by fungi, cyanobacteria, algae, and other types of microbes colonizing the surfaces of cultural heritage objects. Recent studies attempted to assess resistance to QACs in dark-pigmented fungi isolated from stone surfaces (Isola et al. 2021; 2022). Several of the strains isolated (*Acremonium*-like, *Cladosporium* spp., *Exophiala bonariae*, *Aureobasidium pullulans*, *Exophiala oligosperma*, and *Verrucocladosporium dirinae*) displayed high tolerance to QAC-based biocides. Other studies have shed light on the toxic effect of QACs on cyanobacteria and algae (Wu et al. 2021; Qian et al. 2022). Wu et al. (2021) tested alkyl trimethylammonium compounds (ATMAs), a class of QAC cationic surfactants, on two cyanobacteria: *Aphanizomenon ovalisporum* (recently identified as *Chrysosporum ovalisporum*) and *Microcystis aeruginosa*, as well as, two chlorophytes: *Chlorella* sp. and *Scenedesmus* sp. Results showed that cyanobacteria have higher sensitivity to ATMA surfactants than that of green algae. On the former, the growth was inhibited by depression of photosynthesis (via damage to the thylakoid membranes), induction of oxidative stress, and breakage of the cell membrane. Previously, the same authors demonstrated that the octadecyltrimethyl ammonium (ODTMA), a quaternary ammonium cation, imposed inactivation of photosynthesis in cyanobacteria, followed by cell lysis and cellular degradation (Sukenik et al. 2017). On algae, moderate damage to the thylakoid membranes and consequently to the photosynthetic process was observed after treatment with ATMA cations. The greater resistance displayed by the green algae is explained by the authors because they contain an acetolysis-resistant biopolymer, algaenan (Allard and Templier 2000). Qian et al. (2022) revealed the toxic effects and mechanism of a type of benzalkonium chloride on the cyanobacterium *Microcystis aeruginosa*. The quaternary ammonium compound depressed the photosynthetic activities via denaturing-related organelle, caused oxidative damage by producing superoxide radicals, and destroyed the integrity of cell membrane.

Impairments to the photosynthetic system, promoted by the application of two different QAC-based biocides (Biotin T® and Preventol RI80®, at the highest concentration suggested by the producer, i.e., 3% and 2%, respectively), were also studied in the photobiont of the lichen *Xanthoria parietina* (Vannini et al. 2018). These researchers demonstrated the low content of ergosterol in the mycobiont and that the lack of recovery of the lichen 90 days after treatment with the biocide may be related to blockage of the biosynthetic pathway leading to synthesis of this important membrane component. They concluded that the biocides used are effective for eradicating lichen and also produced satisfactory results in preventing re-colonization. However, the persistence of tolerant and resistant forms in stone fissures may promote re-colonization of the rock by lichens and/or fungi, making surface cleaning effective during only a short period (De los Ríos et al. 2012).

## Microbial antagonists

Microbial antagonism occurs as a result of competition between microorganisms for nutrients and space, leading to one microorganism inhibiting growth of the other. It is based on the principle of biological competition (Gause’s law 1934) whereby two different species of bacteria and/or fungi, existing in the same ecological space, cannot coexist in stable equilibrium if they require the same nutrient substrates; one of them, usually the less demanding in terms of nutrition, will become dominant over (and possibly cause extinction of) the other. These procedures can be referred to as “biostabilization techniques,” which are exerted by one species over another, and therefore they do not imply generalized biocidal action, as the final effect is against specific microbial species (Marin et al. 2016). In the field of plant pathology, the use of microbial antagonists to control post-harvest fungal diseases of different fruits and vegetables has been applied since the end of the 1970s, and there has since been a boom in the search for safer and more eco-friendly alternative approaches to biological control (e.g., Dukare et al. 2019; Reyes-Estebanez et al. 2020). In the cultural heritage field, the use of microbial antagonists or biocontrol agents as safe, green biocides as alternatives to the traditional and commercial biocides usually applied to stone-built heritage, although green alternatives may have a toxicological profile similar to their traditional counterparts (see e.g., Silva et al. 2016a), remains very limited. In a recent study, Marin et al. (2016) evaluated two biological biocides on the early twentieth century handmade bricks, using the European standard protocol for evaluation of cleaning methods for cultural heritage (UNI 11551–1:^2014^): Natria (Bayer) a herbicide based on pelargonic acid and that can be used to control weeds on exterior surfaces such as garden paths, and NewFloorCleaner (Chrisal Cleaning Products, Belgium), a product based on probiotics (spores of *Bacillus subtilis*, *Bacillus megaterium*, and *Bacillus pumilus*) and used for disinfecting surfaces in hospitals. The biocides tested are currently not used in cultural heritage conservation, but seem reasonably adequate for this type of use. The study findings suggest that both products can potentially be used as novel biocides on stone surfaces, although Natria has a strong, unpleasant odor and can leave a white patina on the treated surface. Both disappear over time, when the material absorbs water. In addition, at the microscopic level product, Natria left residues that affected the microporosity of the stone, and NewFloorCleaner caused an increase in conductivity and in the concentrations of sulfates and calcium, sodium, and magnesium cations on the treated surface. The authors do not indicate whether these residues disappear over time. In this connection, note the importance of long-term monitoring to know what happens over time, if the residues or aesthetic problems, such as a visible color change, disappear or increase. In general, there is a need for more well-designed long-term experiments to fully explore the dynamic of the materials and developed microbial communities over time.

Important advances have been made in studies carried out in Portugal in the use of bacteria of the genera *Bacillus*, which are capable of producing secondary metabolites with antagonistic activities against fungal isolates that cause biodeterioration on heritage monuments (Caldeira 2021 and references therein). Some strains of *Bacillus subtilis* and *Bacillus amyloliquefaciens* have been reported to produce antifungal peptides. *Bacillus subtilis* is one of the most versatile producers of cyclic lipopeptides, such as surfactin, iturin, and fengycin, amphiphilic membrane-active biosurfactants with potent antifungal activity. These lipopeptides are produced on sporulation of *Bacillus* at the end of the resting stage, which makes bacterial culture a key process in this respect. In addition, Silva et al. (2016b) proposed a simple, rapid method for detecting and characterizing bioactive compounds produced by *Bacillus* strains with high antagonistic activity, particularly against *Penicillium* sp. and *Cladosporium* sp., and to a lesser extent *Alternaria* sp., *Mucor* sp., and *Fusarium oxysporum*. The same authors performed a further study collecting plants such as *Pouteria ramiflora*, known for its antimicrobial, anti-inflammatory, and antifungal activity, and other plants in the families *Apocynaceae* and *Fabaceae* in the Brazilian Cerrado, a tropical highland savanna in the midwestern region of Brazil, in order to extract potentially bioactive compounds (Silva et al. 2019). Furthermore, the same research group has successfully combined the bioactive compounds produced by *Bacillus* sp. CCLBH 1053 and *Pouteria ramiflora* (*Sapotaceae*) extracts as an antifungal agent simultaneously against biodeteriogenic yeast and filamentous fungi (Silva et al. 2019).

The genus *Bacillus* comprises around 380 species with a remarkable potential to produce a large variety of secondary metabolites, including ribosomally and non-ribosomally synthesized antimicrobial peptides. Two terpenes (isoprene and monoterpene α-terpineol) produced by *B. subtilis* exhibit antagonistic activity against cyanobacteria and nematodes (Caulier et al. 2019).

In aqueous ecosystems, bacteria are capable of controlling massive growth of algae and cyanobacteria through physical association or the production of algicidal compounds (Coyne et al. 2022). The aquatic bacterium *Streptomyces neyagawaensis* has been shown to be capable of suppressing the growth of cyanobacteria (such as *Microcystis aeruginosa*) and of a wide range of algae, including *Chlorella* spp. (May et al. 2009). The bacterium *Phaeobacter inhibens* can promote growth of the marine alga *Emiliania huxleyi* at an early stage (mutualistic interaction) but then kill it at a later stage (antagonistic interaction). Bacteria isolated from diatoms of the genus *Pseudo-nitzschia* display algicidal activity, and a few bacteria exhibit lytic activity against both cyanobacteria and microcystins (Coyne et al. 2022).

Different plant parts (roots, stems, flowers, fruit) have traditionally been used to treat some human diseases because they contain several phytochemicals such as flavonoids, alkaloids, tannin, and terpenoids, which possess antimicrobial and antioxidant properties. In the cultural heritage field, in vitro bioactivity testing has been successfully carried out with extracts of spontaneous plants, i.e., *Solanum nigrum* (*Solanaceae* family), *Moricandia arvensis* (*Brassicaceae* family), *Volutaria lippii* (L.), and *Pulicaria inuloides* (both belonging to *Asteraceae* family), which showed strong biocidal activity against bryophytes, green algae, and lichens isolated from two rupestrian churches belonging to a UNESCO World Heritage site in Matera (southern Italy) (Scrano et al. 2020).

## Not all viruses are bad: phages

Bacteriophages (phages), i.e., viruses that infect bacteria, are probably the most abundant life form in the biosphere (Summers 2012; Sadekuzzaman et al. 2015). Viruses have a bad reputation, exacerbated in recent times by the recent global SARS-CoV-2 pandemic. Nevertheless, as stated in an ancient proverb, “The enemy of my enemy is my friend,” and when the bacteria that viruses target are also within our firing range, they become our allies in “viral remediation” or “phage therapy” treatment (Abedon et al. 2017), as long as they are safe for human and environmental health (Soffritti et al. 2019). The potential of phages as antibacterial agents has been known since 1917, i.e., before the discovery of antibiotics by Fleming, in 1928. There is now renewed interest in the use of phages due to the increasing emergence of tolerant and resistant bacteria and to the advantages of phages over antibiotics and chemical agents, especially for inhibiting or disrupting biofilms (Sadekuzzaman et al. 2015), including those formed by *Escherichia coli* (Meng et al. 2011).

Phage therapy has been poorly exploited, despite the fact that it has been demonstrated to be safe and effective, with rapid, simple, and inexpensive phage isolation and production, and highly specific self-replication in the target host bacterium because they work in a key/lock way (Sadekuzzaman et al. 2015). It is sometimes used in the agricultural, food, clinical, and veterinary sectors (e.g., Ni et al. 2020), although infrequently, due to the dominance of antibiotics, which are much more familiar, stable, easy to mass produce and administer, and display broad spectrum efficacy. In addition, the lack of regulatory approval and political aspects has also prevented wide use of phage therapy.

In the cultural heritage field, the few studies involving phages published to date report two different lines of action: (1) phages as protective agents preventing bacterial decay of wood in archaeological sites (Klaassen 2005; Cappitelli et al. 2020) and, (2) eukaryotic viruses (not phages) that infect algae, including the chloroviruses that infect *Chlorella*-like green algae growing on stone surfaces, such as Portland limestone gravestones (Kang et al. 2005; May et al. 2009; Cappitelli et al. 2020). In the first case, the greatest advances have arisen within the framework of the EU project BACPOLES (2002–2005), which is based on the idea that bacteriophages are frequently found in nature, wherever bacteria are present, and that wood-degrading bacteria may already be infected by strain-specific (target) phages. Unfortunately, screening assays aimed at obtaining pure cultures of wood-degrading bacteria and then monoclonal antibodies against bacteria were unsuccessful, and the development of a phage-based wood preservative did not proceed further (Klaassen 2005). In the second case, much of the work on algal viruses has been done in aquatic systems (May et al. 2009). Viruses with antialgal activity have been isolated from sediments, and mature biofilms have been also found on sediment surfaces. However, the aforementioned authors were not able to isolate the viruses from Portland limestone in the laboratory for use in removing biofilms. As in the first case, the laboratory studies were very problematic; the algae grew very slowly and did not reach the required densities in liquid cultures, which, in addition, were frequently contaminated by bacteria and fungi. However, positive results were obtained in laboratory assays using paired algal hosts (*Chlorella* strains, NC64A, Pbi, and Sag 3.83) and viruses (PBCV-1, MT325, and ATCV-1) derived from aquatic systems, confirming the possibility of virus-induced bioremediation to inhibit growth of the alga *Chlorella* on stone. *Chlorella* commonly occurs on historic fountains and other wet stone surfaces (Bolivar-Galiano et al. 2021) and also in lampenflora communities (Nikolić et al. 2021).

Despite the interest in the field of study, to the best of our knowledge, there has been no further progress in this type of research on cultural heritage field for more than 10 years. This may be because despite the advantages of phage therapy (application before, during, or after growth of the host microorganism, high specificity, self-replication of in situ while the host microorganism persists, no reported adverse effects), various limitations and potential side effects have also been described. For example, the EPS matrix and the multi-species or multi-organism nature of the biofilm reduce the effectiveness of treatment, although the first could be overcome by including a mixture of viruses and other substances (like enzymes) that facilitate penetration of the virus through the biofilm matrix. Another drawback is the natural emergence of phage-resistant mutants, which could be minimized by using combined therapies of essential oils (e.g., carvacrol, Ni et al. 2020. Although it should be noted here that the long-term effects of essential oils should be also assessed in relation to their impact on the material, beyond their impact against re-colonization) and other phages. Likewise, the potential release of toxins and the potential incorporation of the virus encoding virulence genes into the host genome could be addressed by using hydrogels (that immobilize the release of toxins, Donlan 2009) and the use of engineered phages lacking virulence genes. Furthermore, the knowledge on environmental bacteria and the role played on cultural heritage is very limited.

## Overlooked strains: pathogens and novel antibiotics

**Fig. 2:**
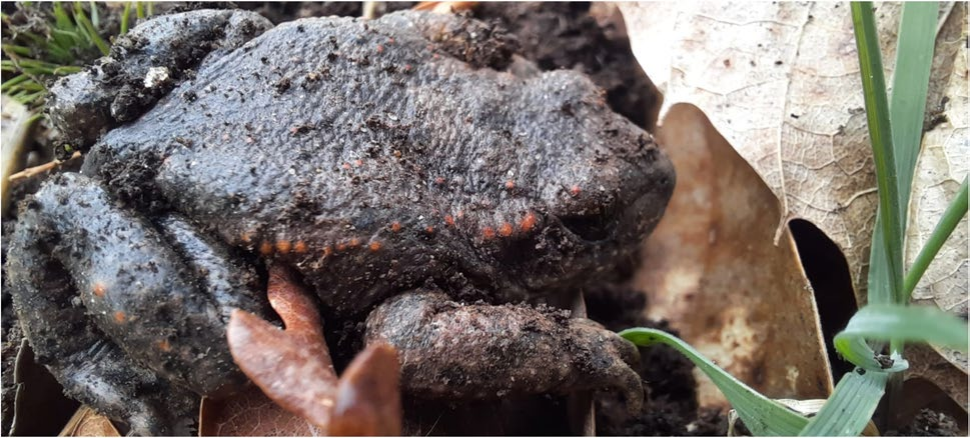
Midwife toad (*Alytes obstetricans*), one of the amphibian species most threatened by chytridiomycosis, is a disease caused by *Batrachochytrium* spp. Photo: Miguel Serrano

Dark and wet heritage sites, such as caves and chapels, can act as reservoirs of pathogenic organisms. In a recent study, Pavlović et al. (2022) detected, for the first time, the dangerous amphibian fungal parasite *Batrachochytrium* on paving stones in a historical European chapel. A high proportion of the massive decline in amphibians worldwide has been associated with this pathogen, and human-mediated translocation of the fungus is the main cause of the widespread distribution of the disease (Rollins-Smith 2020). In Europe, there has been a dramatic decline in some species that are highly susceptible to *Batrachochytrium*, such as the midwife toad (*Alytes obstetricans*, Fig. 2) (Bosch et al. 2020). Microbiological studies of heritage monuments, particularly those using metagenomic tools, should focus on detecting and highlighting the occurrence of emergent pathogens in cultural sites, to prevent making these frequently visited site sources of the spread of hazards either to humans or to other organisms. Along this line, Jurado et al. (2010) focused their review paper on pathogenic microorganisms in caves, such as the human pathogenic black yeast fungi *Exophiala castellanii* and *Exophiala moniliae* found in the Lascaux Caves, as mentioned above. Isolated species of the genera *Amycolatopsis*, *Aureobacterium*, *Brevibacterium*, *Nocardia*, *Nocardioides*, *Rhodococcus*, and *Streptomyces* and members of the family *Micrococcaceae* are responsible for different skin, lung, and brain infections in humans. These microorganisms can also form abscesses that induce sensory, motor, and behavioral disturbances, as well as nausea, headaches, and vomiting. The main opportunistic pathogens of *Nocardia* species include, e.g., *N. farcinica*, *N. nova*, *N. abscessus*, and *N. cyriacigeorgica*, while members of the genus *Gordonia* include *G. bronchialis*, *G. otitidis*, *G. aichiensis*, and *G. terrae*. Regarding fungal strains, there are references to pulmonary histoplasmosis caused by the fungus *Histoplasma capsulatum* found in caves inhabited by bats where it is also common to find the emerging pathogenic fungus *Penicillium marneffei*.

Historically, soil has always been the main source of antibiotics. Thousands of bioactive compounds (e.g., chloramphenicol, tetracycline, and erythromycin) have been extracted from soil bacteria (Cycoń et al. 2019). However, cultural heritage monuments and sites can also be considered as sources of antibiotics. Approximately, two-thirds of all known antibiotics are produced by *Actinobacteria* (a large part of the stone heritage communities), particularly by species of the genus *Streptomyces* (Mast and Stegmann 2018), and many authors consider *Actinobacteria* an inexhaustible source of naturally occurring antibiotics. Historical caves and mines are currently considered emergent sources of novel antibiotics, acting as excellent reservoirs of new species of *Actinobacteria* (Cheeptham and Saiz-Jimenez 2015). Methanotrophic and heterotrophic bacteria found in caves and mines can produce bioactive compounds and may be potential sources of metabolites with antibacterial, antifungal, and anticancer properties. Some examples of drug discovery in caves include the chemical structure of cervimycin A-D; a polyketide glycoside complex obtained from *Streptomyces tendae*, isolated from Grotta dei Cervi (Italy); cyclodysidin D and chaxalactin B produced by a *Streptomyces* sp. isolated from moonmilk (cave milk); and the huanglongmycin A-C complex, synthesized by a strain of *Streptomyces*, found in a cave in China (Martín-Pozas et al. 2020).

## Use of plants to mitigate the effects of climate change on stone heritage

**Fig. 3:**
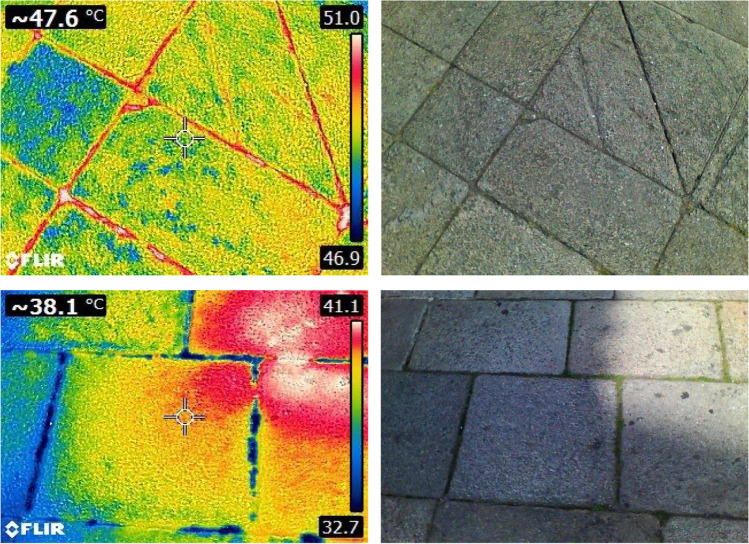
Ecosystem service: urban plant cool granite pavements in Santiago de Compostela (NW Spain), while paving joints filled with cement accumulate the highest temperatures. Photos: Miguel Serrano

Temperature change is a major factor in stone weathering; and therefore, knowing and controlling micrometeorological conditions, i.e., the actual climatic conditions around the stone surfaces, is a determining factor in heritage conservation. Plant landscaping has been suggested to be effective for regulating the cultural heritage microenvironment (Li et al. 2021). Comparison of unshaded areas and areas shaded by trees along the historical Nanjing wall (China) showed that trees could reduce evaporation by 8–18% and prevent the associated risk of deterioration on wall surfaces by buffering freeze–thaw cycles and efflorescences. An ongoing survey, started in the summer of 2022, has shown that small plants growing between paving stones in the historical city of Santiago de Compostela (Galicia, NW Spain), a UNESCO World Heritage City since 1985, can help to counteract the negative effects of global rising temperatures by controlling the biometeorological conditions within the plants (Kevan et al. 2019). As plants cannot move to avoid the effects of hot temperatures, they have evolved mechanisms such as increasing transpiration rates, to cope with the risk of overheating, which ultimately results in maintaining the surrounding temperatures below those on the bare pavement or on paving joints filled with cement (Fig. 3). Despite the small size of these plants and the relatively low cover relative to the whole area (typically less than 15%), the micrometeorological effects of reducing temperatures during heatwaves are noticeable, even at heights of 1.80 m above the pavements (with decreases of up to 3 °C in areas with plants growing between paving stone) contributing to both stone heritage conservation and the habitability of cities (Serrano, preliminary unpublished results). The small plants involved have until now been neglected as weeds, and are routinely removed from cultural sites and historical city centers. The ecological arrangement of these species in the urban fabric (e.g., plants with C4 photosynthetic metabolism, such as *Digitaria* spp. and *Amaranthus* spp., and therefore possessing mechanisms to reduce water loss, prevailing in the more isolated areas) should be studied and used in urban design to enhance the provision of ecosystem services, including micrometeorological temperature control and cohesion of paving stones via lateral root growth.

In addition, climate change is affecting many archaeological sites scattered through forested areas in Europe as fire rates increase (Fernández et al. 2021). A recent study compared the damage, including aesthetic damage, to granite rocks in cultural sites with megalithic and parietal art in areas of the NW Iberian Peninsula characterized by different types of vegetation (Pozo-Antonio et al. 2020); the study findings showed that oak woods could buffer the damaging effects of fire in natural areas, and the authors suggested using oak species as protective belts in conservation planning for cultural sites in natural areas.

## Final considerations and future prospects

In projections for the future of cultural heritage protection, efforts should turn towards the green deal, ecosystem services, and animal and human health, to provide benefits for biodiversity and climate change mitigation and adaptation. This is in accordance with the concept of bioeconomy, as defined by the European Commission, because it involves renewable biological resources and conversion of these into value added products, such as green biocides. It is also consistent with the ONU Sustainable Development Goals (ODS) as the aim is to promote innovative research and sustainable methods of safeguarding cultural heritage (SDG11, goal 11.4) through the use of natural products that replace traditional toxic chemical products and therefore promote the health and well-being of restorers (SDG3, goal 3.9), while also reducing the use of dangerous chemical substances and air, water, and soil pollutants (SDG12, goal 12.4) and promoting sustainable and environmentally friendly industrialization (SDG9).

Although the mechanisms of resistance to QACs in microorganisms that affect cultural heritage remain largely unknown to date, there are studies that explain through them how green algae are less sensitive than cyanobacteria to certain QACs compounds. Furthermore, it can be concluded that the re-colonization promoted by biocides is influenced by factors such as the cleaning process when an effective biocide is correctly applied. It is important to conduct pre-treatment analysis to screen the tolerance to biocides, at least in the strains isolated, and then to use the most appropriate biocide. Microbiological monitoring of the cultural heritage objects after application of biocide treatments would also help to clarify whether the re-colonization is driven by tolerant and resistant microorganisms or by new colonizers, and also to determine the ratios of these two groups. Combining microbiological analysis and culture-dependent approaches will provide a better view of the microbial communities present and to match these with the detection of potential biocide-resistant genes. More complex studies could also focus on the development of multi-strain biofilms in laboratory conditions.

Planning historical urban centers should also promote the growth on pavements of plants that are evolutionarily adapted to cope with increasing temperatures. Furthermore, biological control technologies based on viral remediation or phage therapy is a promising eco-sustainable approach that deserves further consideration in the cultural heritage field, but so far, the results have fallen well short of expectations. Future research lines should aim to confirm the effectiveness of in vivo treatments, the persistence of the effects, and the lack of side effects on real monuments (lack of negative interactions with the substrate). Once these crucial points are elucidated, the methods of application must be optimized and cost evaluation must be conducted, with the aim of developing commercially useful, green conservation products.

## Data Availability

All data generated or analyzed during this study are included in this publication (and the supplementary material).
